# Nitrite reductase activity in F_420_-dependent sulphite reductase (Fsr) from *Methanocaldococcus jannaschii*

**DOI:** 10.1099/acmi.0.000482.v3

**Published:** 2023-04-20

**Authors:** Christian Heryakusuma, Eric F. Johnson, Endang Purwantini, Biswarup Mukhopadhyay

**Affiliations:** ^1^​ Genetics, Bioinformatics, and Computational Biology Ph.D. Program, Virginia Tech, Blacksburg, VA 24061, USA; ^2^​ Department of Biochemistry, Virginia Tech, Blacksburg, VA 24061, USA; ^3^​ Virginia Tech Carilion School of Medicine, Virginia Tech, Blacksburg, VA 24061, USA

**Keywords:** coenzyme F_420_, deazaflavin, F_420_-dependent nitrite reductase (FNiR), F_420_-dependent sulphite reductase (Fsr), F_420_H_2_, methanogen

## Abstract

*
Methanocaldococcus jannaschii* (*Mj*), a hyperthermophilic and evolutionarily deeply rooted methanogenic archaeon from a deep-sea hydrothermal vent, produces F_420_-dependent sulphite reductase (Fsr) in response to exposure to sulphite. This enzyme allows *Mj* to detoxify sulphite, a potent inhibitor of methyl coenzyme-M reductase (Mcr), by reducing it to sulphide with reduced coenzyme F_420_ (F_420_H_2_) as an electron donor; Mcr is essential for energy production for a methanogen. Fsr allows *Mj* to utilize sulphite as a sulphur source. Nitrite is another potent inhibitor of Mcr and is toxic to methanogens. It is reduced by most sulphite reductases. In this study, we report that *Mj*Fsr reduced nitrite to ammonia with F_420_H_2_ with physiologically relevant *K*_m_ values (nitrite, 8.9 µM; F_420_H_2_, 9.7 µM). The enzyme also reduced hydroxylamine with a *K*_m_ value of 112.4 µM, indicating that it was an intermediate in the reduction of nitrite to ammonia. These results open the possibility that *Mj* could use nitrite as a nitrogen source if it is provided at a low concentration of the type that occurs in its habitat.

## Data Summary

The authors confirm all supporting data, code and protocols have been provided within the article.

**Impact Statement**

Nitrite is highly toxic to methanogens and the deep-sea hydrothermal vent environment provides a constant supply of this oxyanion at low concentrations. This environmental situation calls for a nitrite detoxification tool in vent methanogens. Here, the occurrence of F_420_-dependent nitrite reductase (FNiR) activity in F_420_-dependent sulphite reductase (Fsr) opens the possibility of vent methanogens employing this enzyme to detoxify nitrite as well as to generate ammonia for cell biosynthesis.

## Introduction

Nitrite is toxic to methanogens as it inhibits methyl-coenzyme M reductase (Mcr) at low micromolar concentrations [1, 2], an enzyme that is essential for energy production in these archaea [3]. Yet, several *Methanocaldococcus* species and one *Methanotorris* isolate from deep-sea hydrothermal vents and a hot spring thermophilic methanogen utilize nitrate as a nitrogen source [4, 5]. This capability requires the reduction of nitrate to ammonia, which involves nitrite and hydroxylamine as an intermediate; similar to nitrite, hydroxylamine is toxic to many organisms [6, 7]. We therefore looked for the possibility of a nitrite reductase enzyme that would not impart hydroxylamine toxicity in these organisms.

Similar to nitrite, sulphite inhibits Mcr [8, 9] and is toxic to methanogens [4, 10–14]. However, *Methanocaldococcus jannaschii* (*Mj*), an inhabitant of deep-sea hydrothermal vents, is resistant to sulphite and can use this oxyanion as a sole sulphur source [13, 15]. This capability is due to a novel sulphite reductase that uses coenzyme F_420_ as an electron carrier, and is called F_420_-dependent sulphite reductase (Fsr) [15]. The homologues of Fsr are present in all hydrothermal vent methanogens [4, 5]. Phylogenetic and comparative structural analysis has shown that the Fsr homologues form two distinct clades, called FsrI and FsrII, and *Mj*Fsr belongs to the FsrI clade and henceforth we call it *Mj*FsrI [16].

Most sulphite reductases reduce nitrite [15, 17–22] and a preliminary study in our laboratory has shown an indication for this activity in *Mj*FsrI [23]. Also, recently an FsrII from one of the anaerobic methanotrophic archaea (ANME), which oxidize methane anaerobically, has been shown to act as an F_420_-dependent nitrite reductase (FNiR) and lacks sulphite reductase activity [17]. Accordingly, we examined the nitrite reductase activity of *Mj*FsrI and characterized the respective kinetic properties. The ecological relevance of the findings was also explored.

## Methods

### Purification of *Mj*FsrI

The protein was purified anaerobically from *Mj* cells grown with sulphite as a sulphur source via a previously reported procedure [15, 24] but with the following modifications. Cell extracts were fractionated via precipitation with ammonium sulphate and gravity flow-based column chromatography conducted at room temperature (~25 °C) and inside an anaerobic chamber filled with a mixture of N_2_ and H_2_ (96 : 4, v/v). The (NH_4_)_2_SO_4_ and NaCl solutions were prepared in 25 mM potassium phosphate buffer, pH 7 (buffer A). All chromatography resins were obtained from Cytiva, except F_420_-Sepharose which was prepared in the laboratory [15, 25, 26]. Recovery of the enzyme was followed by use of an assay for F_420_-dependent sulphite reductase (Fsr) as described below. The first step of purification was treatment of an *Mj* cell extract with (NH_4_)_2_SO_4_ at 60 % saturation, and activity was found in the supernatant, which was fractionated over a 1×20 cm phenyl-Sepharose column with 6 ml resin. After sample application, the column was washed with five solutions of (NH_4_)_2_SO_4_ at the following concentrations, and these were applied in the sequence as presented: 1, 0.75, 0.5, 0.25 and 0 M. The volume of each wash was 12 ml, except that of the first (1 M) which was 36 ml. The eluates from the 0.25 M (NH_4_)_2_SO_4_ wash contained Fsr activity and these were pooled and loaded onto an F_420_-Sepharose column [15, 25, 26] (1×10 cm; 4 ml resin). After sample application, the column was washed first with 28 ml of buffer A and then five aliquots of 8 ml NaCl solutions at the following NaCl concentrations: 0.1, 0.2, 0.3, 0.4 and 0.5 M. Most of the activity was found in the 0.3 M NaCl fractions, which were pooled. The resulting preparation was fractionated on a 1×20 cm column packed with 6 ml QAE-Sephadex resin. Then, the column was washed with 36 ml of buffer A and 12 ml aliquots of four solutions with NaCl at the following concentrations: 0.25, 0.5, 0.75 and 1 M. The 0.25 M NaCl fractions contained Fsr activity and were pooled. SDS-PAGE showed that this final preparation contained an apparently homogeneous enzyme preparation.

### Enzyme activity and protein assays and SDS-PAGE

The assays for FNiR, Fsr and F_420_H_2_-dependent hydroxylamine reductase were performed anaerobically using reduced F_420_ (F_420_H_2_) as the electron donor and respective electron acceptors, which were nitrite, sulphite and hydroxylamine, following a previously described procedure [15, 17, 27]. Briefly, this procedure involved spectrophotometric monitoring of the oxidation of F_420_H_2_ at 400 nm, and an extinction coefficient value of 25 mM^−1^ cm^−1^ for F_420_ was used to calculate the reaction rate [28]. The standard assay employed a reaction mixture with the following components in a total volume of 0.8 ml, and all assays were initiated by enzyme addition: 100 mM potassium phosphate buffer, pH 7; 40 µM F_420_H_2_; 500 µM sodium nitrite or 1 mM sodium sulphite or 500 µM hydroxylamine. In the assays that were used to determine the kinetic constants [15], the concentration of the relevant substrate was varied.

### Iron and acid-labile sulphur content determination

The contents of iron and acid-labile sulphur of *Mj*FsrI were determined as described previously [17], except a solution containing 71.8 µg ml^−1^ protein in 25 mM potassium phosphate buffer, pH 7, was used. The ammonia produced in the Fsr reaction was estimated using a glutamate dehydrogenase-based assay [15, 17, 29] employing an AA0100 Kit (Sigma-Aldrich).

### UV–visible spectroscopy and HPLC analysis of the flavin component of *Mj*FsrI

The UV–visible spectrum of a 300 µl anaerobic solution of *Mj*FsrI containing 21 µg of homogeneous protein in 25 mM potassium phosphate buffer, pH 7, and 250 mM NaCl was obtained at 25 °C using a Beckman Coulter DU800 spectrophotometer as described previously [17].

The type of flavin present in *Mj*FsrI was identified and its amount was determined via non-degradative extraction followed by HPLC analysis at room temperature via an established method [17], but with modifications. A 400 µl solution of 28.7 µg purified protein in 25 mM potassium phosphate buffer, pH 7, was used and the filtered extract was concentrated by evaporation under a flow of nitrogen before analysis.

## Results

**Fig. 1.**
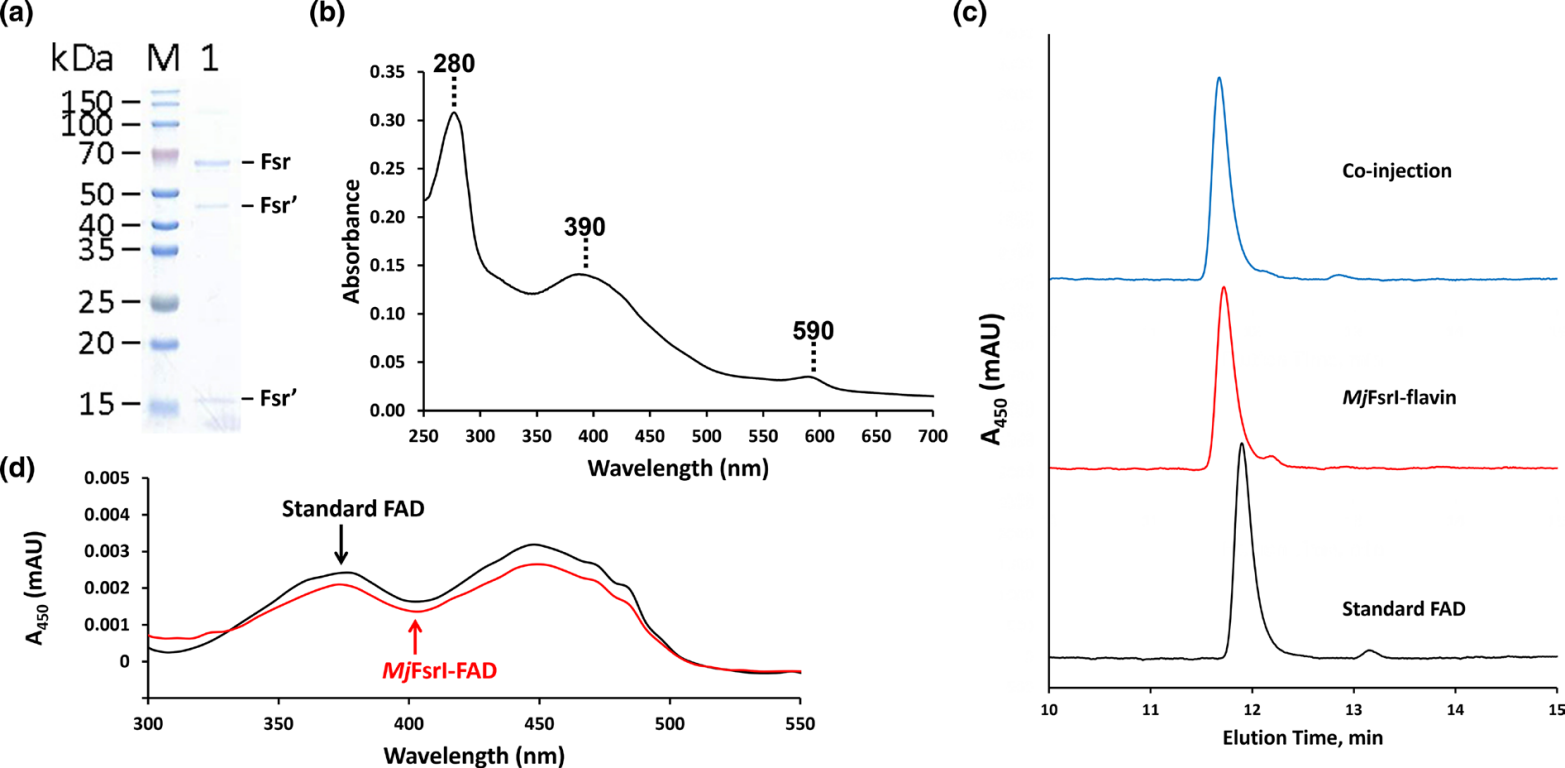
Subunit size, UV–visible spectroscopic characteristics and prosthetic groups of *Mj*FsrI. (a) SDS-PAGE profile of purified *Mj*FsrI. Fsr (intact *Mj*FsrI), ~69 kDa; Fsr′ (degradation products of *Mj*FsrI), ~45 and ~16 kDa. (b) UV–visible spectrum of *Mj*FsrI obtained under anaerobic conditions. (c) Reversed-phase HPLC analysis of a methanol–methylene chloride extract of *Mj*FsrI. Standard FAD, analysis of a 100 µl volume of 0.5 µM flavin adenine dinucleotide (FAD); *Mj*FsrI-flavin, analysis of a 100 µl methanol–methylene chloride extract from 28.7 µg homogeneous protein; Co-injection, analysis of a 100 µl 1 : 1 (v/v) mixture of a 0.5 µM solution of FAD and methanol–methylene chloride extract of *Mj*FsrI. (d) UV–visible spectrum of *Mj*FsrI’s flavin cofactor as collected by the diode array detector of an HPLC unit.

Before embarking on investigating new activity of *Mj*FsrI, we examined the purified *Mj*FsrI for the core properties that were established at the time of its discovery [15]. In SDS-PAGE, the protein exhibited three bands at ~16, ~45 and ~69 kDa (Fig. 1a), where the last value matched the theoretical subunit size of *Mj*FsrI, which is 69.79 kDa. The ~16 and ~45 kDa bands are known to originate from the degradation of this protein during the sample preparation for SDS-PAGE [15]. The purified enzyme displayed a UV–visible spectrum typical of sirohaem in a low-spin ferric state with peaks at 280, 390 and 590 nm (Fig. 1b) [15, 30]. Following this validation, we investigated the new properties of the protein.

### Structural and spectroscopic characteristics of *Mj*FsrI

Reversed-phase HPLC identified the flavin extracted from *Mj*FsrI as FAD (Fig. 1c, d). It was estimated that *Mj*FsrI carried 0.52 mol of this molecule per mole of subunit, which suggested that a dimer of the protein assembled one FAD molecule. Chemical assays showed that *Mj*FsrI held 23.81±1.16 mol iron and 23.92±2.49 mol acid-labile sulphur per subunit, indicating that it assembled six [Fe_4_-S_4_] clusters. This value is consistent with the recently available crystal structure of *Mj*FsrI [31].

### Nitrite reduction activity in *Mj*FsrI and kinetics of the reaction

**Fig. 2.**
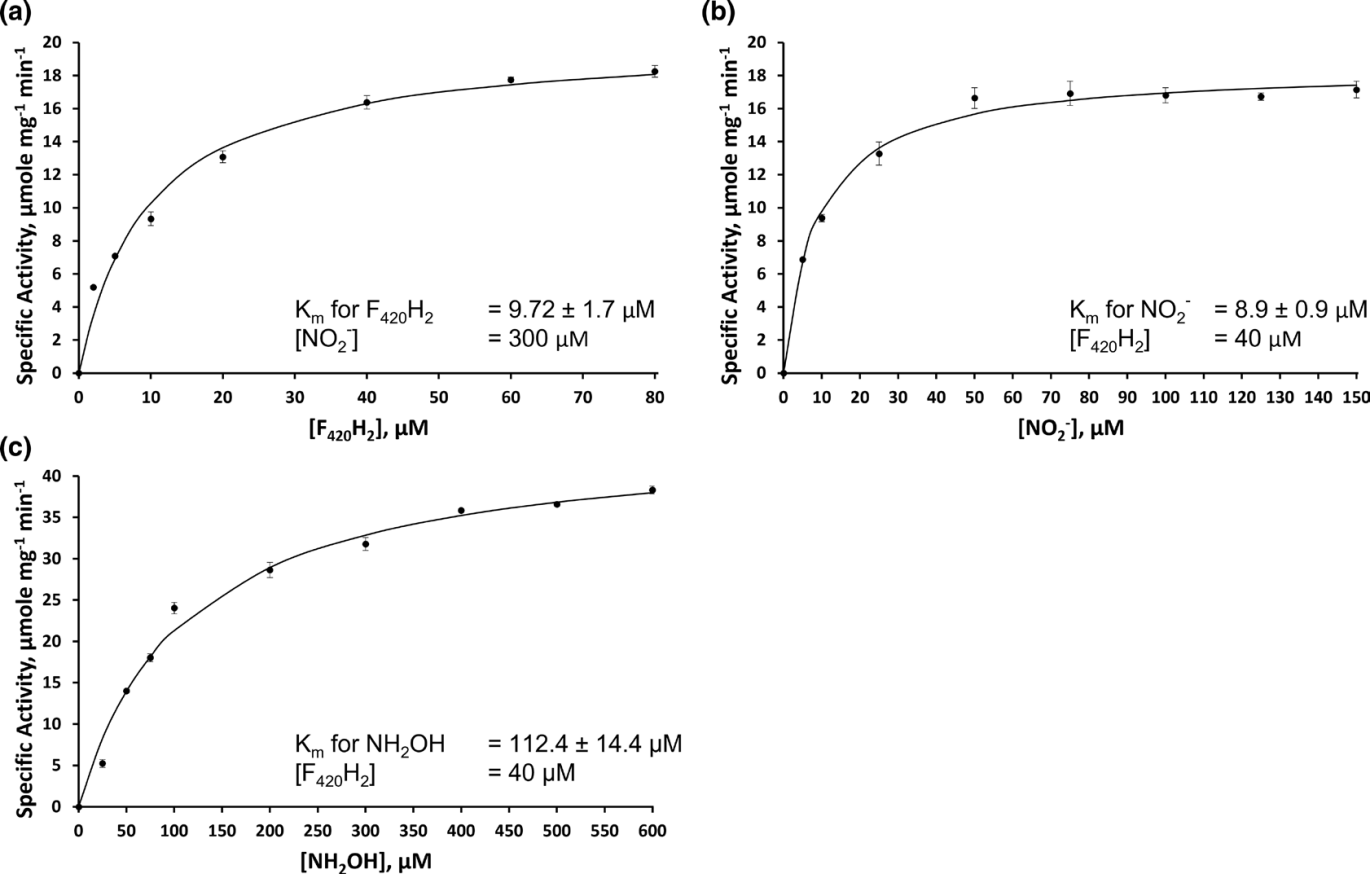
Kinetic analysis of the *Mj*FsrI reaction. Specific activity is defined as the amount (μmol) of F_420_H_2_ oxidized per minute per milligram enzyme. Substrate for which the concentration was varied: (a) F_420_H_2_; (b) nitrite; (c) hydroxylamine. Each data point is an average of values from three independent assays and the respective standard deviations are shown. Each solid curve represents the best fit of the data to the Henry–Michaelis–Menten hyperbola function, *v*=*V*_m_[S]/*K*_m_+[S]; fitting was performed by using the add-in Solver analysis tool in Microsoft Excel [39].

*Mj*FsrI catalysed the reduction of nitrite and hydroxylamine to ammonia by utilizing F_420_H_2_ as an electron donor and was not able to utilize NADH or NADPH for these conversions. Accordingly, we term this action as FNiR activity. At a fixed concentration of 300 µM for nitrite and a concentration range of 2–80 µM for F_420_H_2_, the apparent *K*_m_ for F_420_H_2_ was 9.72±1.7 µM and the apparent maximum velocity (*V*_m_) value was 20.3±0.96 µmol F_420_H_2_ oxidized or 40.6±1.92 µmol electrons transferred per minute per milligram enzyme (Fig. 2a). Similarly, with 40 µM F_420_H_2_ and 5–150 µM nitrite, the apparent *K*_m_ for nitrite was estimated to be 8.9±0.9 µM and the *V*_m_ was 18.4±0.36 µmol of F_420_H_2_ oxidized or 36.8±0.72 µmol electrons transferred per minute per milligram enzyme (Fig. 2b). A kinetic analysis at 40 µM F_420_H_2_ and 25–600 µM hydroxylamine showed that the apparent *K*_m_ for hydroxylamine was 112.4±14.4 µM and the respective *V*_m_ was 45.3±1.9 µmol of F_420_H_2_ oxidized or 90.6±3.8 µmol electrons transferred per minute per milligram enzyme (Fig. 2c). From an average of the values for the amounts of products in three independent 30 min FNiR reactions with *Mj*FsrI, the enzyme produced 0.0427±0.0005 µmol F_420_ and 0.0133±0.0051 µmol ammonia in an assay with 0.08 µmol F_420_H_2_ and 0.40 µmol nitrite; in effect, 0.043 µmol F_420_H_2_ was consumed or 0.086 µmol electrons was made available for nitrite reduction. Since the production of 1 mol nitrite to ammonia would require 6 mol electrons or 3 mol F_420_H_2_, and the enzyme produced F_420_ and ammonia at a ratio of 3 : 1, in the FNiR reaction, about 94 % of the consumed reducing equivalents was recovered in the product.

## Discussion

Fsr of *Mj* exhibited FNiR activity. This observation could be rationalized thermodynamically and it is also consistent with the chemical mechanism established for other sulphite reductases. Since F_420_H_2_ with a mid-point redox potential (*E* ^0^′) value of −360 mV (see equation 1 below) is an effective reductant for Fsr-catalysed sulphite reduction (equations 2 and 4), thermodynamically the enzyme would be able to utilize F_420_H_2_ for nitrite reduction (equations 3 and 5). Studies with *Archaeoglobus fulgidus* (*Af*) dissimilatory sulphite reductase (Dsr) have shown that the transformation of sulphite to sulphide involves the formation of enzyme-bound SO_2_^−^ and SO^−^ as intermediates which are stabilized by a set of basic residues (Arg^98^, Arg^170^, Lys^211^ and Lys^213^) [21, 32]. The SO_2_^−^ intermediate is structurally similar to NO_2_^−^ and *Af*Dsr indeed reduces nitrite using the above-mentioned structural units [21, 32]. The Arg^98^, Arg^170^, Lys^211^ and Lys^213^ of *Af*Dsr are fully conserved in *Mj*FsrI (Arg^355^, Arg^423^, Lys^460^ and Lys^462^ [15, 31]) and therefore *Mj*FsrI employed a common chemical mechanism for reducing sulphite and nitrite; the recently available crystal structure of *Mj*FsrI indeed shows that Arg^355^, Arg^423^, Lys^460^ and Lys^462^ are involved in the binding of sulphite and two water molecules at the active site of this protein [31]. The ability of *Mj*FsrI to reduce hydroxylamine (NH_2_OH) indicated that the Fsr-catalysed reduction of nitrite proceeded through the intermediate formation of hydroxylamine and this sequence is also seen with the other sulphite/nitrite reductases [17, 18, 20, 21]. The enzyme was efficient in the utilization of the F_420_H_2_-derived reducing equivalents as it exhibited almost 100 % recovery of this resource into ammonia, the product. These data indicated that NH_2_OH, a reaction intermediate, did not accumulate and this finding is consistent with the observation that the rate of hydroxylamine reduction was more than double of that for the overall nitrite reduction reaction. The higher *K*_m_ value for hydroxylamine did not pose a problem for this conversion as this intermediate was probably not released from the enzyme.

(1) $$F_{420}+2e^{-}+2H^{+}\to F_{420}H_{2}\;\;\;\;\;\;\;E^{0^{\prime }}=-360\;mV$$

(2) $$HSO_{3}^{-}+6e^{-}+6H^{+}\to HS^{-}+3H_{2}O\;\;\;\;\;\;\;E^{0^{\prime }}=-116\;mV$$

(3) $$NO_{2}^{-}+6e^{-}+8H^{+}\to NH_{4}^{+}+2H_{2}O\;\;\;\;\;\;\;E^{0^{\prime }}=+440\;mV$$

(4) $$HSO_{3}^{-}+3F_{420}H_{2}\to HS^{-}+3H_{2}O+3F_{420}\;\;\;\;\;\;\;\Delta G^{0^{\prime }}=-135\;kJ/mol$$

(5) $$NO_{2}^{-}+3F_{420}H_{2}+H^{+}\to NH_{4}^{+}+2H_{2}O+3F_{420}\;\;\;\;\;\;\;\Delta G^{0^{\prime }}=-457\;kJ/mol$$

The *K*_m_ value of *Mj*FsrI for nitrite (~9 µM) was comparable to a value that has been reported for sulphite (12 µM) and a similar case was found for the specific activities of this enzyme (μmol electrons transferred min^–1^ mg^–1^): 37 for nitrite reduction (this study) and 32 for sulphite reduction [15]. Thus, the nitrite reduction activity of *Mj*FsrI could be physiologically relevant in *Mj* with *in vivo* roles in nitrite detoxification and deriving nitrogen nutrition from this oxyanion. We elaborate on these two possibilities below.

As mentioned in the Introduction, *Mj*FsrI acts as a sulphite detoxification enzyme and allows *Mj* to use this toxic compound as a sulphur source [13, 15]. Similar to sulphite, nitrite is highly toxic to methanogens as it oxidizes the Ni(I) centre of coenzyme F_430_, a prosthetic group of Mcr [1], an essential enzyme for energy production in these organisms [3]; at a concentration of 50 µM, nitrite fully inactivates purified Mcr in 15 min [1]. In the natural habitat of *Mj*, a segment of a deep-sea hydrothermal vent, mixing of extremely hot and anaerobic vent water with oxygen-containing cold seawater brings the temperature to a level that is conducive for living organisms [33]. At this locale, the nitrite concentration is kept at a very low level due to chemical reoxidation to nitrate [34]. However, for a constant albeit low-level supply of this oxyanion and high sensitivity of Mcr towards it, it would be advantageous for a vent methanogen, such as *Mj*, to carry a nitrite detoxification tool, and with the observed high activity and low *K*_m_ value for nitrite (~ 9 µM), *Mj*FsrI could satisfy this need.

The highest nitrite concentration in a deep-sea hydrothermal vent is never more than 4 µM [34, 35]. Based on the data in Fig. 2b, at this nitrite concentration, *Mj*FsrI will provide an FNiR activity of about 6 µmol F_420_H_2_ oxidized per minute per milligram protein, which would translate to an ammonium production rate of 2 µmol min^–1^ mg^–1^ protein. The doubling time of *Mj* is about 26 min [36], which corresponds to a maximum growth rate of 1.6 h^−1^ or 1.6 g of daughter cells per 1 g of mother cell mass per hour. Also, nitrogen constitutes about ~15 % of the cell mass [37]. Thus, to produce 1.6 g of new cells per hour, 1 g of mother cells needs to generate 0.24 g of usable nitrogen or 0.29 g or 0.017 mol ammonia per hour. As estimated from the published data (Fig. 2a of [15]), under sulphite induction, Fsr constitutes about 10 % of the total cell proteins; 50 % of the dry weight of a cell is due to proteins [38]. This means that 1 g of mother cells could contain up to 50 mg of Fsr protein, and consequently, could produce 0.006 mol ammonia per hour. Therefore, in a hydrothermal vent environment, the FNiR activity of *Mj*FsrI would allow *Mj* to utilize available nitrite as a sole nitrogen source to maintain about one-third of its maximal growth rate as determined in a laboratory [36] if the nitrogen is the only limiting nutrient. A similar role has also been proposed recently for FsrII in an ANME from marine methane seep sediment [17]. Thus, the Fsr group broadly protects Mcr of marine methanogens and certain ANMEs from sulphite and nitrite inhibition and provides sulphur and nitrogen nutrition to these organisms by reducing these oxyanions.

In summary, the protein that has been described as *Mj*FsrI [15] was shown to provide an FNiR function with physiologically relevant kinetic properties without causing toxicity of hydroxylamine, a reaction intermediate. A calculation based on the kinetic properties of the enzyme, growth kinetics of the organism, and known concentrations for nitrite in the hydrothermal vent fluid showed that the FNiR activity of *Mj*FsrI would support a reasonably high growth rate for *Mj* with nitrite as the sole nitrogen source if it is induced under *in situ* conditions [15]. This assessment made for a rather low concentration of nitrite, a toxic oxyanion for the methanogens, sets the stage for growth studies with *Mj* and other vent methanogens with nitrite as the sole nitrogen source in a continuous culture system. Here a steady supply of nitrite at a low concentration would provide an ecologically relevant environmental condition.
